# Protein tyrosine phosphatase 4A3 (PTP4A3/PRL-3) promotes the aggressiveness of human uveal melanoma through dephosphorylation of CRMP2

**DOI:** 10.1038/s41598-019-39643-y

**Published:** 2019-02-28

**Authors:** Laura Duciel, Océane Anezo, Kalpana Mandal, Cécile Laurent, Nathalie Planque, Frédéric M. Coquelle, David Gentien, Jean-Baptiste Manneville, Simon Saule

**Affiliations:** 1 Institut Curie, PSL Research University, CNRS, INSERM, Orsay, France; 2 Université Paris Sud, Université Paris-Saclay, CNRS, INSERM, Orsay, France; 3 Institute for Medicine and Engineering, University of Pennsylvania, Philadelphia, 19104 USA; 4 CALYM LYSARC Institut Carnot, Paris, France; 5 Université Paris Diderot, Sorbonne Paris Cité, France; 6 Institut Curie, PSL Research University, Translational Research Departement, Genomics Platform, Paris, France; 7 Institut Curie, Paris Sciences and Letters Research University, CNRS, Paris, France

## Abstract

Uveal melanoma (UM) is an aggressive tumor in which approximately 50% of patients develop metastasis. Expression of the PTP4A3 gene, encoding a phosphatase, is predictive of poor patient survival. PTP4A3 expression in UM cells increases their migration *in vitro* and invasiveness *in vivo*. Here, we show that CRMP2 is mostly dephosphorylated on T514 in PTP4A3 expressing cells. We also demonstrate that inhibition of CRMP2 expression in UM cells expressing PTP4A3 increases their migration *in vitro* and invasiveness *in vivo*. This phenotype is accompanied by modifications of the actin microfilament network, with shortened filaments, whereas cells with a inactive mutant of the phosphatase do not show the same behavior. In addition, we showed that the cell cytoplasm becomes stiffer when CRMP2 is downregulated or PTP4A3 is expressed. Our results suggest that PTP4A3 acts upstream of CRMP2 in UM cells to enhance their migration and invasiveness and that a low level of CRMP2 in tumors is predictive of poor patient survival.

## Introduction

Uveal melanoma (UM) is the most common intraocular tumor in adults. This tumor is very aggressive and up to 50% of patients subsequently develop distant metastasis, predominantly in the liver^1–3^. Most patients survive less than 12 months after metastasis occurrence and currently there is no effective treatment to cure metastatic UM, or to improve the overall survival^4^. The genome-wide analysis and gene expression profiling (GEP) have allowed the identification of two major categories of UM, characterized by a low (class1) or a high (class2) metastatic potential^5^. A recent study based on a comprehensive multiplatform analysis of 80 UMs subdivided these two categories into four subgroups with different genomic alterations, transcriptional features, somatic copy number, and DNA methylation profiles^6^.

We previously showed that the strong expression of protein tyrosine phosphatase 4A3/protein of regenerating liver-3 (PTP4A3/PRL-3) is predictive of metastasis using a transcriptomic approach^7^. Moreover, we showed that PTP4A3 expression in UM cells increases their migration *in vitro* and invasiveness *in vivo*^7^. PTP4A3 is overexpressed in many types of cancers, including gastric cancers^8^, ovarian cancers^9^, non-small cell lung cancer^10^, human gliomas^11^, nasopharyngeal carcinoma^12^, metastatic colorectal carcinomas (CRCs), breast cancer, and cervical cancer^13^. In addition, PTP4A3 is not only a marker of poor prognosis, but is also involved in oncogenesis and is a key metastatic event^14^. We previously showed that PTP4A3 promotes aggressiveness through membrane accumulation of matrix metalloproteinase 14 (MMP14)^15^. Indeed, MMP14 belongs to the transmembrane metalloproteases (MMPs), a class of molecules that are responsible for the degradation and turnover of the extracellular matrix (ECM), an event that is required for cell migration and invasiveness. Among PTP4A3 substates there are currently three cytoskeletal proteins that have been identified as PTP4A3 targets, providing strong evidence of PTP4A3 role promoting migration and invasion by cytoskeleton remodeling^16–18^.

Here, we have identified CRMP2 as a new target of PTP4A3 by 2D phosphoprotein analysis. These two proteins interact in co-immunoprecipitation assays. We found that CRMP2 is less phosphorylated on T514 after interaction with PTP4A3 in comparaison to the catalytically inactive mutant of the phosphatase, PTP4A3 (C104S).

CRMP2 is a cytosolic phosphoprotein that interacts with numerous binding partners to affect microtubule dynamics, protein endocytosis, and vesicle recycling^19^. CRMP2 acts largely by stabilizing polymerized tubulin at the plus end of microtubules^20^. Several distinct signaling pathways regulate CRMP2 phosphorylation on different amino acids, to change CRMP2-protein binding interactions. Modifications of the phosphorylation state of CRMP2 change its activity. Indeed, phosphorylated CRMP2 loses its affinity for microtubule heterodimers, thus reduces microtubule stability. In addition to tubulin, CRMP2 binds to the cytoskeletal proteins actin and vimentin^19^.

In the present study, we demonstrate that PTP4A3 expression or CRMP2 downregulation affect microrheological properties by increasing the stiffness of UM cells. This suggests that CRMP2 downregulation has an effect similar to that of CRMP2 dephosphorylation. We showed that CRMP2 loss in UM cells overexpressing PTP4A3, affects the organization of the actin network, increased MMP14 accumulation at the cell membrane, increases the migration *in vitro* and invasiveness *in vivo*. CRMP2 is downregulated in PTP4A3 expressing UM tumors.

## Results

### PTP4A3 expression reduces phosphorylation of CRMP2 on T514

**Figure 1:**
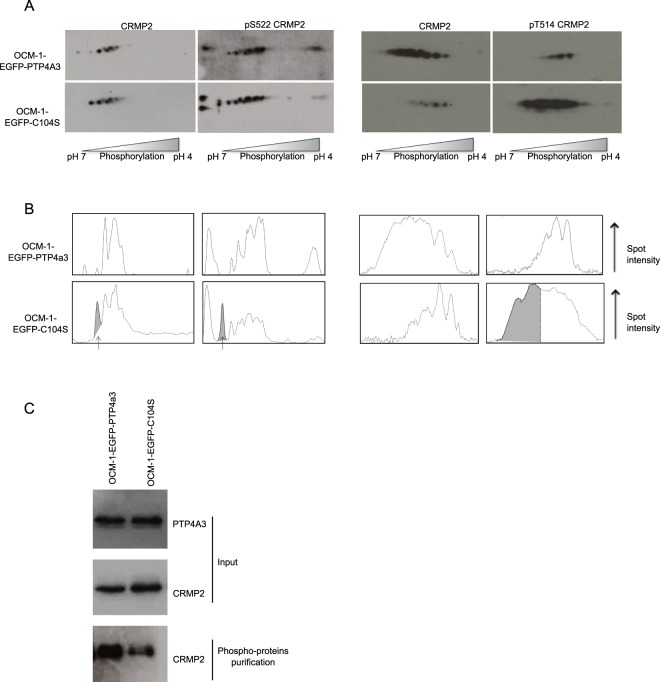
PTP4A3 expression reduces CRMP2 phosphorylation. (**A**) The phosphorylation state of CRMP2 was compared between OCM-1-EGFP-PTP4A3 and OCM1-EGFP-C104S cells by 2D gel electrophoresis. After Western blotting, detection was performed using an anti-CRMP2 antibody that recognizes total CRMP2 or phospho-specific anti-CRMP2 (S522) and anti-CRMP2 (T514) antibodies. (**B**) Spot densitometric profiles of phosphorylated CRMP2 were generated using ImageJ64. The grey peaks indicate those present for OCM1-EGFP-C104S cells which are absent from OCM1-EGFP-PTP4A3 cells. (**C**) Western blot analysis of total lysates (input) and lysates after phosphoprotein purification as loading controls.

We previously showed that stable PTP4A3 expression promotes invasion of OCM-1 UM cells^7^. Here, we identified phosphoproteins regulated by PTP4A3 by performing 2D phosphoprotein gel analysis between OCM-1 cells expressing wildtype PTP4A3 and those expressing the phosphatase inactive C104S mutant of PTP4A3, devoid of a pro-invasive effect^7^. This was followed by mass-spectroscopy of the differential spots, which identified CRMP2 among other proteins. CRMP2 is a phosphoprotein that generates more than seven spots in 2D gel electrophoresis^21^. Figure 1A shows the profile of CRMP2 spots by 2D electrophoresis of OCM-1 cells expressing EGFP-PTP4A3 or EGFP-C104S using anti-CRMP2, anti-CRMP2 S522, and anti-CRMP2 T514 antibodies. The densitometric profiles of CRMP2 (Fig. 1B) show that the number of CRMP2 phosphorylated spots were reduced in the presence of wildtype PTP4A3, especially those of T514, whereas there is more total phosphorylated CRMP2 in these cells (Fig. 1C), possibly due to the overexpression of SRC kinases in PTP4A3-overexpressing cells^22^.

### PTP4A3 and CRMP2 interact

**Figure 2:**
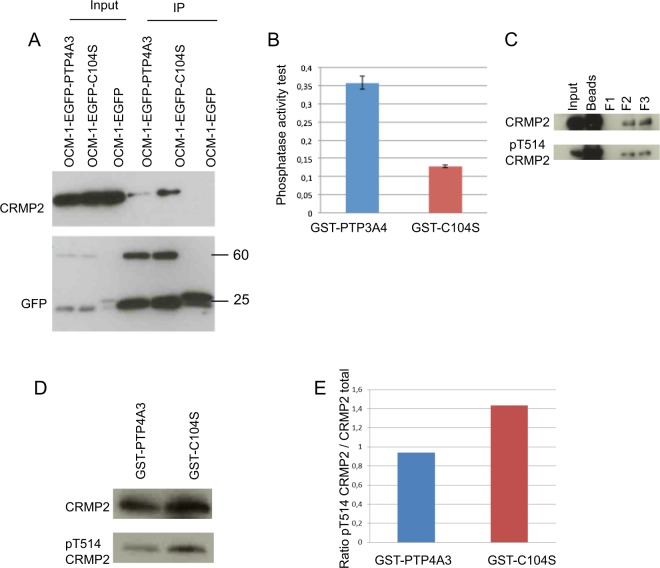
PTP4A3 and CRMP2 interact. (**A**) Coimmunoprecipitation of PTP4A3 and CRMP2 using a GFP-Trap_A kit. This kit allows the immunoprecipitation of GFP recombinant proteins from OCM-1-EGFP, OCM-1-EGFP-PTP4A3, and OCM-1-EGFP-C104S cells. GFP recombinant protein and binding partners were revealed by Western blotting with anti-CRMP2 and anti-GFP antibodies. Input: 10% of the loading. (**B**) Phosphatase activity test of GST fusion proteins. The dephosphorylation capacity of GST-PTP4A3 or GST-C104S was assessed by incubation with OMPF substrate and measuring the absorbance at 450 nm. (**C**) Immunoprecipitation of CRMP2 from OCM-1 cells. After immunoprecipitation, CRMP2 was eluted in three successive fractions. The presence of CRMP2 and its phosphorylation state (T514) was verified by Western blotting. (**D**) GST pull-down experiment. Purified phosphorylated CRMP2 was incubated with GST-PTP4A3 and GST-C104S. After the pull-down, the ability of CRMP2 to bind GST fusion proteins was assessed by Western blotting with an anti-CRMP2 antibody and the phosphorylation state of CRMP2 with an anti-CRMP2 (T514). (**E**) Quantification of CRMP2 T514 phosphorylation by analysis of the densitometric profiles of the bands using ImageJ64. Band intensity is represented by the peak area. The ratio of peak area of CRMP2 to that of CRMP2 (T514) was determined.

PTP4A3 and CRMP2 would be expected to interact if CRMP2 is a target of PTP4A3. Thus, we carried out co-immunoprecipitation assays using the GFP-Trap^®^ kit. Cell lysates were prepared from OCM-1 cells expressing EGFP-PTP4A3, EGFP-C104S, or EGFP, and GFP-Trap^®^ beads were used for immunoprecipitation. The input and precipitated proteins were then separated by SDS-PAGE and analyzed by Western blotting (Fig. 2A). CRMP2 was detected only in cells expressing either EGFP-PTP4A3 or the mutant EGFP-C104S after co-immunoprecipitation. Much less CRMP2 was recovered from cells expressing wildtype PTP4A3 than that expressing the C104S mutant, yet the mutated phosphatase was designed to increase the retention time with interacting partners, suggesting that the interaction between CRMP2 and the phosphatase is direct.

To study whether the dephosphorylation of CRMP2 mediated by PTP4A3 is direct we performed a GST pull down experiment with GST-PTP4A3 and GST-C104S fusion proteins. Determination of the phosphatase activity of GST-PTP4A3 and GST-C104S, tested *in vitro* using the OMFP substrate (Fig. 2B), showed that GST-PTP4A3 can dephosphorylate the OMFP substrate, leading to higher absorbance at 450 nm than for GST-C104S. CRMP2 was immunoprecipitated from OCM-1 cell lysates, eluted in three successive fractions, and the presence of phosphorylated T514 CRMP2 analyzed by Western blotting (Fig. 2C). Fractions F2 and F3, containing phosphorylated T514 CRMP2, were used for subsequent pull downs.

Fusion proteins and phosphorylated CRMP2 were mixed with glutathione-Sepharose beads. After incubation and pull down, a Western blot was performed with anti-CRMP2 and anti-CRMP2 (T514) antibodies and the ratio of the detected proteins determined. CRMP2 incubated with active phosphatase exhibited a reduced amount of phosphorylated T514 (Fig. 2D,E), favoring the hypothesis of CRMP2 as a direct target of PTP4A3.

### CRMP2 downstream of PTP4A3 reduces cell migration and invasiveness

**Figure 3:**
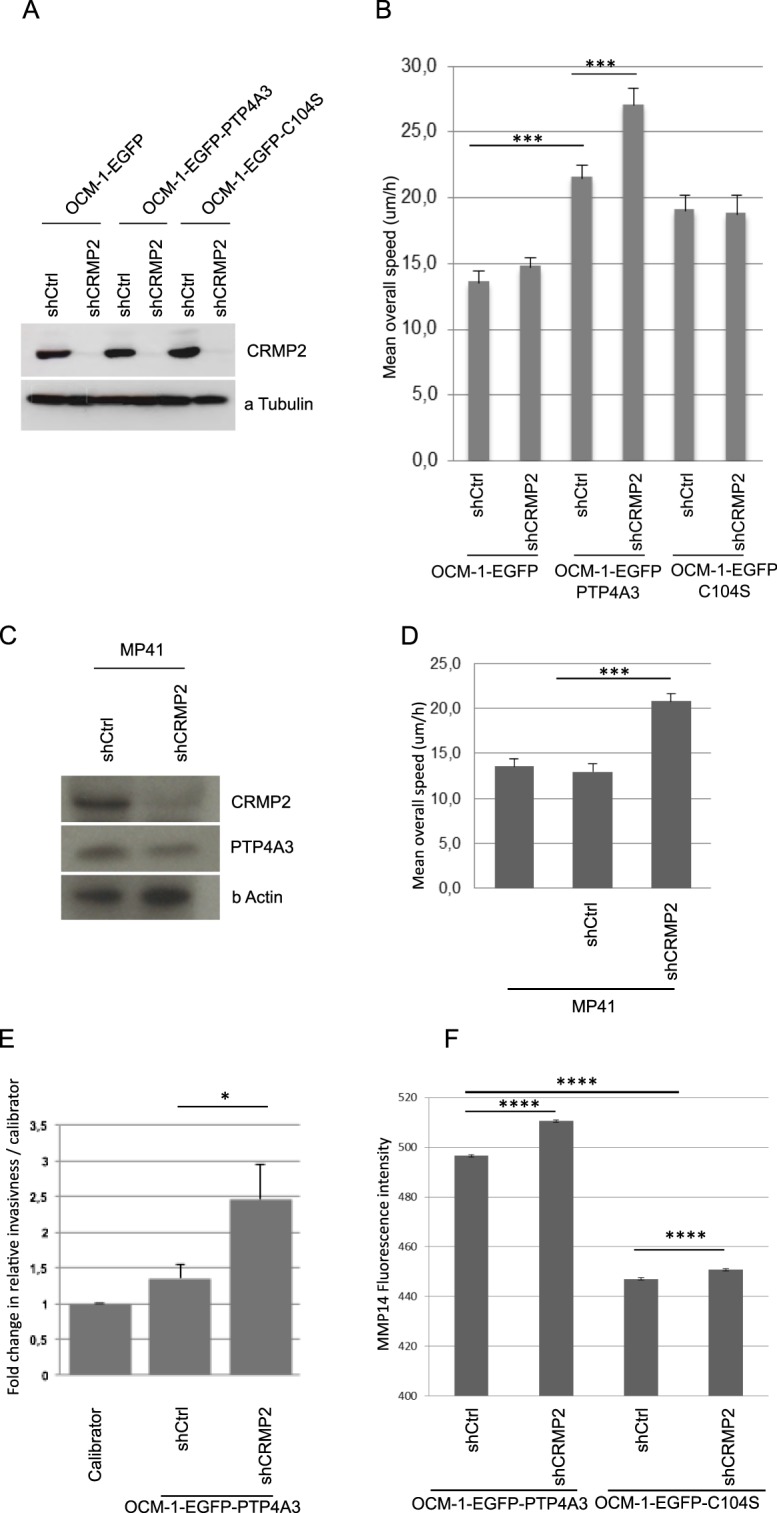
CRMP2 expression downstream of PTP4A3 reduces cell migration and invasiveness. (**A**) Western blot showing the knockdown of CRMP2 in OCM-1 cells expressing EGFP, EGFP-PTP4A3, or EGFP-C104S. Twenty micrograms of protein extract was loaded. The detection of α-tubulin was performed as a loading control. (**B**) The mean overall speed of OCM-1 cells was assessed by a 2D random cell migration assay by time-lapse video microscopy. Data are shown as the mean values +/− SD. ~100 cells were tracked per condition. ***p < 0.001, Student’s t-test. (**C**) Western blot showing the knockdown of CRMP2 in human PDX-MP41 cells and endogenous expression of PTP4A3. Twenty micrograms of protein extract was loaded. The detection of β-actin was performed as a loading control. (**D**) The mean overall speed of MP41 cells was assessed by a 2D random cell migration assay by time-lapse video microscopy. Data are shown as the mean values +/− SD. ~100 cells were tracked per condition. ***p < 0.001, Student’s t-test. (**E**) Quantitative analysis of the relative invasiveness of EGFP-PTP4A3shCtrl and EGFP-PTP4A3shCRMP2 cells. Cells (0.25 × 10^6^) were inoculated into the CAM of chick embryos and the presence of human cells assessed by real-time PCR analysis of chick GAPDH and human alu sequences in the chick femur DNA. Values for the calibrator were arbitrarily defined as 1. The graphs show the means +/− SD. *P < 0.05 (embryos n = 14/cell line), Student test. (**F**) MMP14 localization on the cell surface assessed by flow cytometry of nonpermeabilized OCM-1 cells. n > 8000 cells analyzed, (****p < 0.0001), Student test.

To determine whether the function of CRMP2 is required for the PTP4A3-associated pro-migratory and invasive phenotypes of UM cells, we performed RNA interference experiments by lentivirus shRNA anti-CRMP2 infection (Fig. 3A). Random cell migration on collagen I and invasiveness by the CAM (Chorioallantoic membrane) *in vivo* dissemination assay were tested. The downregulation of CRMP2 expression significantly increased the migration speed of EGFP-PTP4A3 expressing cells, whereas the migration speed of the control cells expressing EGFP-C104S or EGFP was not affected (Fig. 3B). We performed the same knock-down experiment on human PDX-MP41 cells (Fig. 3C,D), which exhibit a low metastatic genetic profile^23,24^ and a moderate level of endogenous PTP4A3 expression (Fig. 3C). The migration speed of PDX-MP41 cells increased significantly after CRMP2 knock-down (Fig. 3D). In addition, downregulation of CRMP2 expression increased PTP4A3-associated invasiveness *in vivo* (Fig. 3E). These findings suggest that CRMP2 antagonizes PTP4A3-associated migration *in vitro* and *in vivo* invasiveness of UM cells. In addition, we observed that OCM-1-EGFP PTP4A3 exhibited an increased number of micronuclei when compared to the OCM-1-EGFP C104S mutant and this number significantly increased after CRMP2 knock down (Supplementary Fig. 1). Similar results of micronuclei increased after CRMP2 knockdow was observed with de PDX-MP41. This is reminiscent of the observation of Mazouzi *et al*., 2016 showing that replication stress induced phosphorylation of CRMP2 regulates genome stability^25^.

### CRMP2 affects MMP14 membrane accumulation

CRMP2 may affect cell migration and invasiveness due to its role in vesicular trafficking^19^. PTP4A3 increased MMP14 at the cell surface^15^, therefore, we quantified endogenous MMP14 membrane localization in nonpermeabilized OCM-1 cells by flow cytometry. The expression of PTP4A3 increased the quantity of MMP14 localized at the cell surface, as observed previously. Furthermore, the downregulation of CRMP2 expression in EGFP-PTP4A3 expressing cells significantly increased the membrane level of MMP14 relative to that of the shRNA control (Fig. 3F), suggesting that CRMP2 may be involved in MMP14 vesicular transport by negatively regulating its accumulation at the cell surface. Thus, the role of CRMP2 in the migration and invasion of UM cells may be explained, in part, by the control of MMP14 levels at the membrane.

### CRMP2 affects cytoskeleton

Although CRMP2 is known to bind to actin, little is known, in non neuronal cells, about its role in the regulation of the actin network. In neurons, CRMP2 and CRMP4 form complexes to bridge microtubules and actin and thus work cooperatively to regulate growth cone development and axonal elongation^26^. Phosphorylation by Rho kinase modulate CRMP-2 localization on actin filaments^27^.

**Figure 4:**
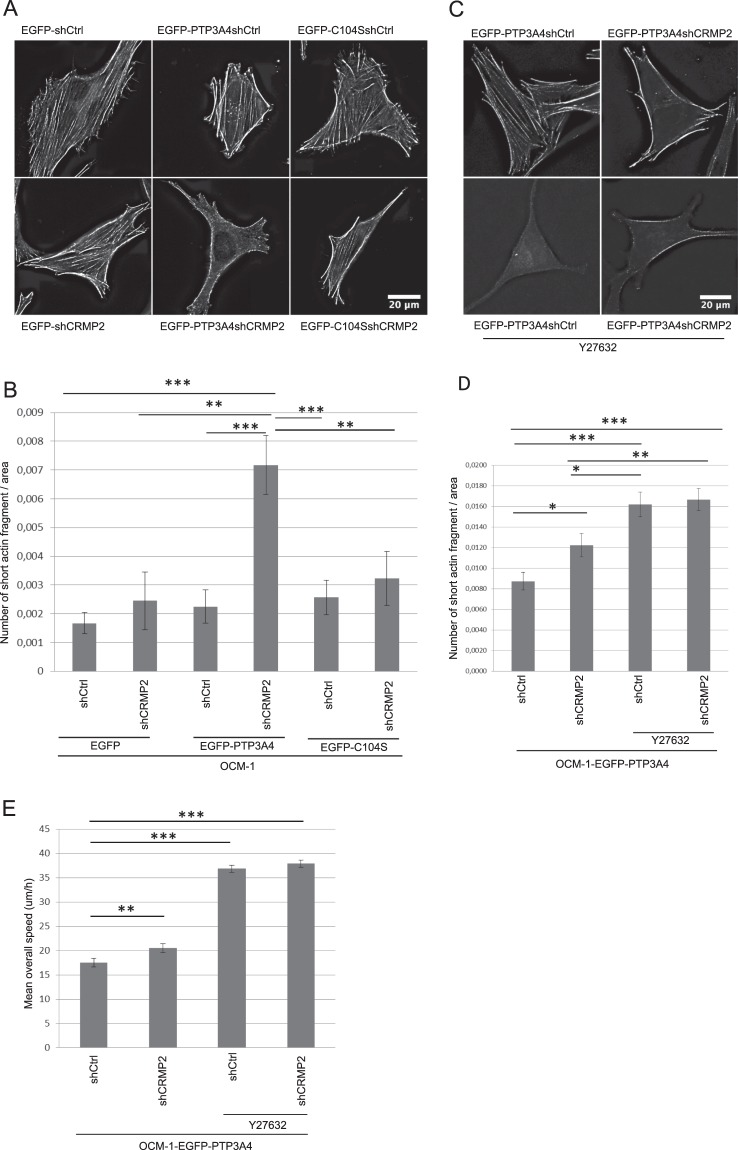
CRMP2 affects the actin network. (**A**) Visualization of the actin cytoskeleton network in OCM-1 cells as assessed by Phalloïdin staining. After deconvolution, images were processed using ImageJ64. Scale bar = 20 μm. (**B**) Quantification of the number of fragments of < 40 adjoining pixels normalized to the cell area in OCM-1 cells. Data are shown as the mean values +/− SEM. ~20 cells were analyzed. ***p < 0.001, **p < 0.01, Student’s t-test. (**C**) Visualization of the actin cytoskeleton network in OCM-1 cells as assessed by Phalloïdin staining of cells treated with Y27632 for 24 h. After deconvolution, images were processed using ImageJ64. Scale bar = 20 μm. (**D**) Quantification of the number of fragments of < 40 adjoining pixels normalized to the cell area in OCM-1 cells treated or not with Y27632. Data are shown as the mean values +/− SEM. ~60 cells were analyzed. ***p < 0.001,*p < 0.01, *p < 0.05, Student’s t-test. (**E**) The mean overall speed of OCM-1 cells treated with Y27632 as assessed by a 2D random cell migration assayed by time-lapse video microscopy. Data are shown as the mean values +/− SD. ~100 cells were tracked per condition. ***p < 0.001, **p < 0.01, Student’s t- test.

Therefore, we analyzed modifications of the actin network following CRMP2 knock-down in OCM-1 cells expressing EGFP-PTP4A3, EGFP-C104S, or EGFP. The actin network in the cells was visualized by phalloïdin staining (Fig. 4A) and the number of short actin fragments quantified and normalized by the cell area (Fig. 4B). All cells showed a similar actin network, with the presence of numerous stress fibers, except those expressing PTP4A3 in which CRMP2 expression was knocked down. Indeed, there was a significant reduction in the number of actin stress fibers in these cells and thus an increase in the number of short actin fragments in these cells (Fig. 4B). This observation may be surprising, given that stress fibers are often described as contractile bundles necessary for cell migration. Yet, here the strong reduction in the number of stress fibers correlates with increased cellular migration. However, several studies have shown that stress fibers are more prominent in stationary cells^28^ and that these structures are ill-suited for cellular motility.

As ROCKII is involved in stress-fiber formation and was showed to phosphorylate CRMP2^27^, we assessed the actin network in OCM-1 cells treated with a ROCKII inhibitor (Y27632) to confirm our results. Cells treated with Y26732 had much fewer stress fibers compare to non-treated cells (Fig. 4C and D), confirming the role of ROCKII in stress-fiber formation. We then analyzed the migratory capacity of these cells. Cells treated with Y27632 migrated faster in comparaison to non-treated cells, independently of CRMP2 expression (Fig. 4E), which confirms that the increase cellular migration is associated with less stress fibers in OCM-1 cells. As Cofilin phosphorylation is regulated by ROCKII *via* LIMK, we also confirmed the dephosphorylation of Cofilin S9 in Y27632-treated cells by Western blotting (Supplementary Fig. 2).

The role of CRMP2 on microtubule dynamics is well characterized in neurons^19^. We also analysed the effect of CRMP2 knock-down on microtubule dynamics in OCM-1 cells expressing EGFP-PTP4A3 or EGFP (Supplementary Fig. 3). Cells were treated with nocodazole, a microtubule depolymerizing agent, washed, and then fixed at different time. We performed a tubulin immunofluorescence on fixed cells in order to follow microtubule repolymerization. The results show that 5 minutes after nocodazole treatment, PTP4A3 shCRMP2 expressing cells have shorter microtubules compare to the other cells (Supplementary Fig. 3B) which suggest a delay in microtubule repolymerization. This result is correlated with a decrease of the percentage of cells with an aster (Supplementary Fig. 3C). Thus, CRMP2 regulated microtubule polymerization in UM cells as it was known in neurons. Interestingly, the effect of CRMP2 knock-down on microtubules are PTP4A3 dependant in these cells.

### CRMP2 affects the microrheological properties of cells

**Figure 5:**
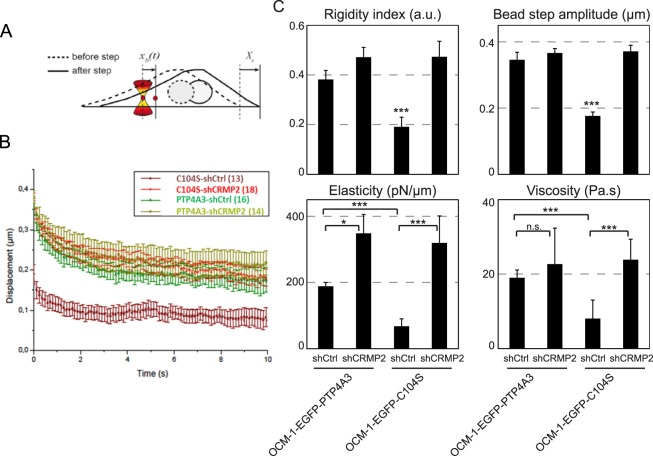
CRMP2 affects the microrheological properties of the cells. (**A**) Sketch of the microrheology experiments. A 2-µm-diameter bead internalized in the cell is trapped with an optical tweezer. At time *t* = 0 s, the microscope stage is moved in a *X*_*s*_ = 0.5-µm step displacement. After the initial rapid displacement of the bead from the trap center, the bead position *x*_*b*_(*t*) relaxes towards the center of the optical trap, which acts as a spring. Single particle tracking of the bead allows determination of the viscoelastic relaxation curves shown in B. (**B**) Average bead displacement curves showing viscoelastic relaxation of the bead towards the trap center following a 0.5-µm step displacement of the microscope stage for OCM-1-EGFP-PTP4A3 and OCM1-EGFP-C104S cells treated with shCtrl or shCRMP2. (**C**) Quantification of the relaxation curves using a phenomenological model-independent approach (upper graphs) yielding the rigidity index and the bead-step amplitude, and the Standard Linear Liquid (SLL) viscoelastic model (lower graphs) yielding the elasticity and viscosity of the cytoplasm. Data were obtained from N = 16 and 14 beads for the OCM-1-EGFP-PTP4A3 cells treated with shCtrl or shCRMP2, respectively, and from N = 13 and 18 beads for OCM1-EGFP-C104S cells treated with shCtrl or shCRMP2, respectively. Error bars represent the standard error. p-values were determined using Student’s t-test for unpaired samples (***p < 0.001, *p < 0.05).

The cytoskeleton maintains cell architecture, generates force and contractility, and plays an active role in many cellular processes, such as cell division and cell migration. Actin filaments and microtubules have been shown to contribute to intracellular mechanics^29^ and CRMP2 participates in regulating the cellular architecture through its interaction with cytoskeletal proteins. We thus performed microrheology experiments (Fig. 5A) to determine the viscoelastic properties of the cell cytoplasm. Viscoelastic relaxation experiments showed that the rigidity of OCM-1 C104S cells increased after CRMP2 knock down to a level already obtained in OCM-1 PTP4A3 cells in the presence of CRMP2 (Fig. 5B). Quantification of the relaxation curves using a phenomenological model-independent approach showed a significant increase in the rigidity index and bead-step amplitude in OCM-1 C104S cells upon CRMP2 knock-down (Fig. 5C, upper panels). In contrast, the knock-down of CRMP2 in OCM-1 PTP4A3 cells did not modify intracellular rigidity, suggesting that knocking down CRMP2 was equivalent to dephosphorylation of the protein by PTP4A3 for this property. Consistent with these results, analysis of the relaxation curves using the Standard Liquid Linear (SLL) viscoelastic model showed that knocking down CRMP2 induced a significant increase in both elasticity and viscosity in OCM-1 C104S cells, but only a slight increase in elasticity in OCM-1 PTP4A3 cells (Fig. 5C, lower panels).

### Effect of CRMP2 expression on the survival of patients with uveal melanoma

**Figure 6:**
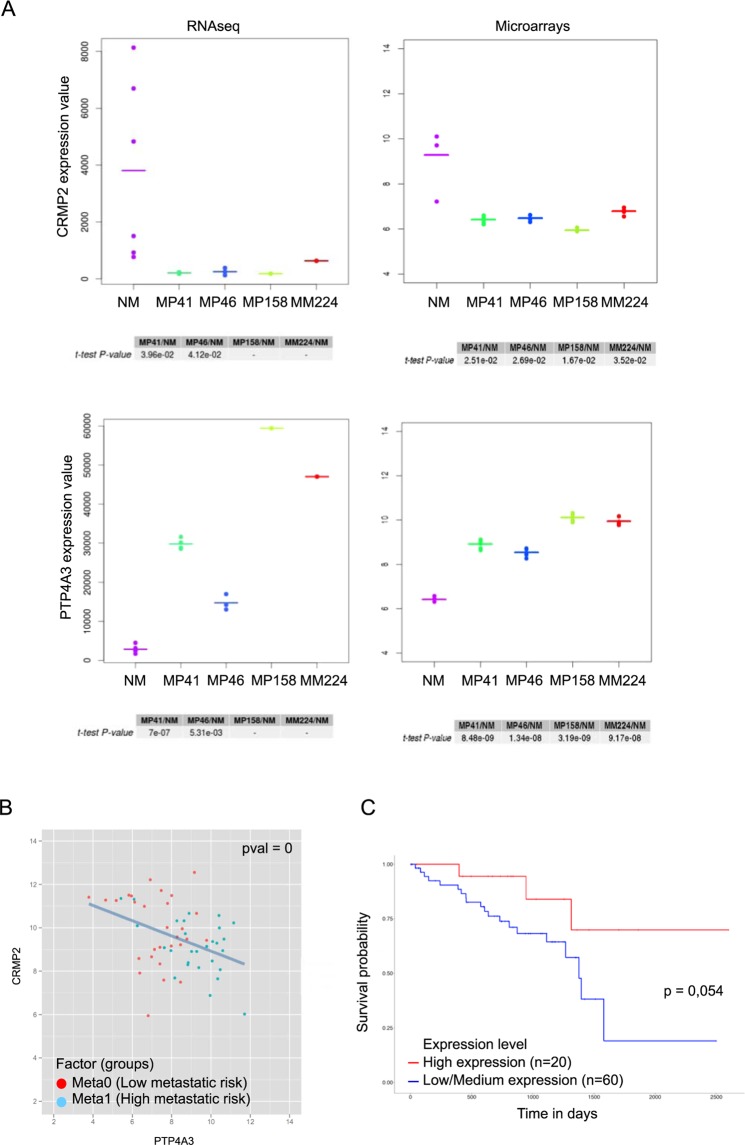
Expression level of PTP4A3 and CRMP2 in normal melanocytes, uveal melanoma cell lines, tumors and survival probability in patients. (**A**) A Transcriptome analysis was conducted on replicates of normal melanocytes (NM), and PDX of primary tumors such as MP41, MP46, MP158, and a PDX of a metastasis MM224. Normal melanocytes, and uveal melanoma tumor cells from cell sorting of dissociated PDX were analyzed on Affymetrix Human Transcriptome Array v2.0 and on Illumina to performed gene expression analysis^48^. Signal from microarray data were normalized according Genosplice’s pipeline based on a RMA normalization step as described previously^49^. RNAseq signals are Deseq. 2-Normalized counts processed by Genosplice’s pipeline too^50^. A paired t-test was applied between UM and normal melanocytes. In our comparisons, CRMP2 is down regulated in UM vs NM, as observed in RNASeq and in microarray datasets. PTP4A3 is upregulated in UM vs normal melanocytes. No p-value are calculated for MP158 and MM224 in RNAseq dataset because a unique sample was sequenced contrary to first experiments done on microarrays. MP41 and MP46 were described previously^23,24^. MP158 and MM224 are GNAQ mutated and BAP-1 mutated. (**B**) PTP4A3 and CRMP2 expression were determined by microarrays analysis. Specimens were analyzed on GeneChip Human Genome U133 Plus 2.0 microarrays (Affymetrix) as described previously^7^. PTP4A3 and CRMP2 expression are inversely correlated in tumors (Spearman coefficient of −0,4). Spearman instead of Pearson correlation, because the Spearman correlation is less sensitive than the Pearson correlation to strong outliers. 63 tumors were analysed, each one divided in two groups: the red dots correspond to meta0 tumors (low metastatic risk) and blue dots to meta1 tumors (high metastatic risk)^7^. (**C**) Effect of CRMP2 expression on UM survival. Kaplan-Meier analysis of CRMP2 expression in 80 uveal melanoma tumors from the TCGA database (http://ualcan.path.uab.edu/index.html).

We tested the expression level of CRMP2 and PTP4A3 in UM cell lines, as well as in primary cultures of normal uveal melanocytes. Two types of measures giving similar results were used (Fig. 6A). The results showed that the normal melanocytes^30^ expressed higher levels of CRMP2 and lower levels of PTP4A3 than any of the UM cell lines.

CRMP2 mRNA expression in patient tumors inversely correlated with PTP4A3 mRNA expression (Spearman coefficient of −0,4) and Kaplan-Meier survival plots using CRMP2 as a binary variable showed that CRMP2 levels below the median were associated with reduced survival (P = 0.054) (Fig. 6B,C).

## Discussion

UM cells that overexpress PTP4A3 migrate faster *in vitro* and are more invasive *in vivo* compare to cells expressing the catalytically inactive mutant PTP4A3(C104S), implicating the phosphatase activity of PTP4A3 in the metastatic process.

Here, we identified CRMP2 as a new target of PTP4A3 by 2D phosphoprotein analysis (Fig. 1). Indeed, CRMP2 was less highly phosphorylated, especially on T514 (Fig. 2E). However, purification of the phosphoproteins showed phosphorylated CRMP2 to be more abundant in cells expressing PTP4A3 compare to those expressing the mutant (C104S). This contra-intuitive observation may be surprising, but a phosphoproteomic study showed that PTP4A3-expressing cells show a pronounced increase in protein tyrosine phosphorylation, especially due to Src activation^22^. Thus, it is possible that PTP4A3 expression increases global CRMP2 phosphorylation, and more particularly those of tyrosine (CRMP2 has at least six tyrosine phosphorylation sites: Y32, Y251, Y275, Y431, Y479, and Y499)^31^ while locally decreasing CRMP2 phosphorylation of T514. Our results also suggest that there is a physical interaction between CRMP2 and PTP4A3 and that this interaction leads to decreased CRMP2 phosphorylation on T514, suggesting that CRMP2 is a direct target of PTP4A3. Moreover, the greater recovery of CRMP2 from cells carrying the C104S mutant than those carrying wildtype PTP4A3 in GFP-trap experiment suggests a higher retention time of the mutated phosphatase, reinforcing this possibility. Glycogen synthase kinase-3β (GSK-3β) phosphorylates CRMP2 at T514 and controls axon/dendrite fate by promoting neurite elongation via microtubule assembly^32^. The phosphorylation of CRMP2 T514 by GSK-3β inhibits CRMP2 activity, leading to microtubule depolymerization and destabilization. The role of microtubules in cell migration is well known and the microtubule depolymerizing drug colcemid^33^ reduces cell migration. Another study also showed that microtubule depolymerization interferes with cell motility^34^.

Our study strongly suggests that CRMP2 acts to brake the migration and invasion process of UM cells (Fig. 3). However, the increase in the speed of cells in which CRMP2 was knocked down was only observed in those expressing PTP4A3. CRMP2 knockdown did not change the speed of migration of cells expressing the mutant (C104S) or GFP alone. Along this line, CRMP2 knockdown was shown to decrease the duration of closure in a scratch wound assay based on PDGF-induced cell migration^35^. Thus, the presence of PTP4A3 is necessary to reveal the loss of the effect of CRMP2. Our previous studies showed that PTP4A3 regulates integrin β1 in adhesion structures during the migration of UM cells^36^ and promotes aggressiveness through membrane accumulation of MMP14^15^. Thus, PTP4A3 appears to have a large spectrum of action, with many targets, that drives cell invasiveness. Loss of CRMP2 increased the number of micronuclei in PTP4A3-expressing cells. This increase in micronuclei formation is reminiscent of the importance of CRMP2 phosphorylation to prevent genome instability^25^ and PTP4A3 may be instrumental to increase genome instability favoring transformation progression of these tumors. We also showed that CRMP2 affect MMP14 membrane accumulation and given the role of CRMP2 in vesicular trafficking^37^, we can hypothesis that PTP4A3 drives MMP14 membrane accumulation, and therefore invasion, in part by regulating CRMP2. MMP14 membrane accumulation is also observed in cells expressing the mutant C104S, but the level reached in these cells is not sufficient to increase cell migration. Another major player involved in the oncogenesis of UM *via* vesicular trafficking is the small GTPase ARF6. ARF6 activates multiple pathways, including PLC/PKC, Rho/Rac, and YAP^38^. ARF6 has also been shown to regulate the exocytosis of MMP14 in breast cancer invasion^39^ and PTP4A3 and ARF6 have been shown to interact in co-immunoprecipitation assays^40^. Thus, the coordination of all these players in UM metastasis needs to be investigated.

Another mechanism that can explain the role of CRMP2 in migration and invasion is cytoskeleton remodeling. Indeed, we also show that more rapidly migrating OCM-1 cells contain fewer stress fibers (Fig. 4). This observation was confirmed by the increase of migration capacity of cells treated with the ROCKII inhibitor, Y27632. However, the increase in the migration speed of ROCKII-inhibited cells was not dependent on CRMP2.

**Figure 7:**
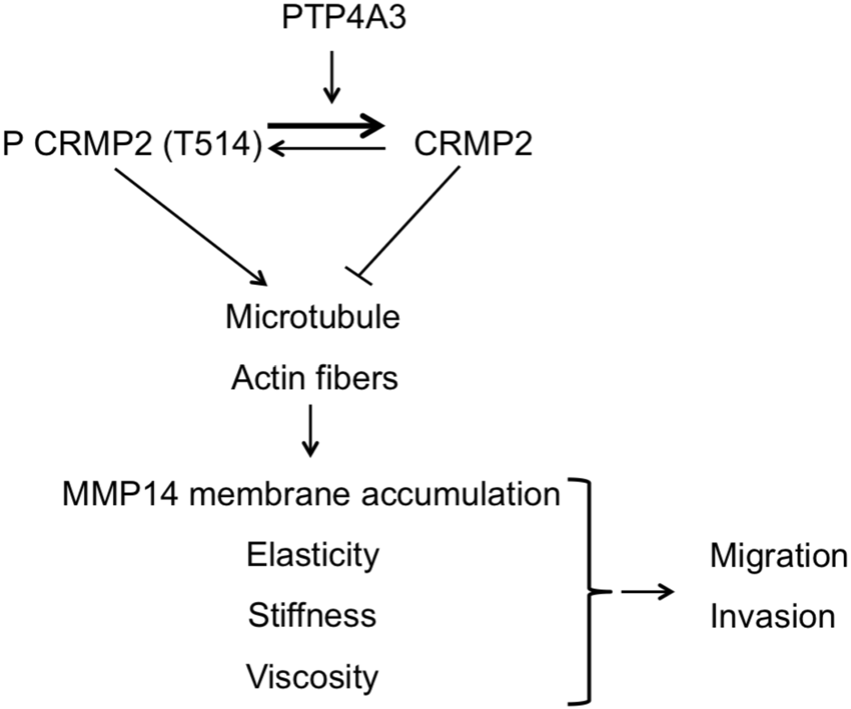
Model for the regulation of PTP4A3 and CRMP2 expression in uveal melanoma cells. The phosphatase PTP4A3 dephosphorylates CRMP2 on the T514 affecting cytoskeleton (microtubule function and actin fibers formation) which affect cells micro-rheoligical properties and MMP14 membrane accumulation leading to an increase of both migration and invasion. Arrow: activation, blind-ended arrow: inhibition.

The experiments on the microrheological properties of the cells show that knocking down CRMP2 is equivalent to the effect of PTP4A3 on the protein in controlling intracellular rigidity (Fig. 5). The lower rigidity of the cytoplasm of C104S cells is consistent with the reported microtubule destabilization in these cells. However, these microrheological modifications do not explain the migration/invasion phenotype, as knocking down CRMP2 in C104S cells changed the elastic properties of the cells but had no effect on the motility properties, suggesting additional key properties of PTP4A3 in UM invasion. The Fig. 7 shows a proposed model of CRMP2 regulation by PTP4A3 in uveal melanoma cells. This model suggests that the phosphatase PTP4A3 dephosphorylates CRMP2 on the T514 affecting microtubule function and actin fibers formation. These modifications on cytoskeleton will affect cells micro-rheological properties and MMP14 membrane accumulation leading to an increase of both migration and invasion.

The level of CRMP2 expression is a good prognostic factor for UM patients and is predictive of metastasis formation. The role of CRMP2 in cancer has not been extensively studied, but several studies have been performed on the use of CRMP2 as a prognostic marker. A study carried out in breast cancer showed a lower level of CRMP2 in cancer cells than in healthy tissue, whereas there is greater phosphorylation of CRMP2 at T509, S518, and S522^41^. In contrast, some studies have shown the opposite, with a higher CRMP2 level associated with tumoral progression^42,43^. Thus, the role of CRMP2 on tumor development appears to vary depending on the cell type.

Finally, many clinical trials are currently testing drugs that inhibit CRMP2 phosphorylation, as CRMP2 is involved in various neurological disorders (Alzheimer’s disease, schizophrenia, cephalic pain, etc.)^19^. This is notably the case of (S)-Lacosamide, used in preclinical models of cephalic pain^44^. However, our results, suggesting increased invasion after CRMP2 dephosphorylation, should encourage additional studies to verify the safety of CRMP2 dephosphorylation, especially concerning the metastasis development in UM.

## Materials and Methods

### Cell culture and shRNA

We previously established OCM-1 clones stably expressing EGFP-PTP4A3, EGFP-C104S, or EGFP^7^. Human PDX-MP41 cells bearing the GNA11 mutation and wild-type BAP1, disomic for chromosome 3^23^ were maintained in RPMI (Gibco) with 20% FBS (HyClone UK laboratories). CRMP2 shRNA lentiviral particles were purchased from Santa Cruz Biotechnology (sc-44485-V). Cells were transduced with three specific lentiviral constructs targeting CRMP2 or control non-target shRNA (sc-108080). UM cells expressing shRNA were selected with puromycin.

### Antibodies and constructs

Rabbit polyclonal anti-CRMP2 (CP2161) and rabbit polyclonal anti-CRMP2 (S522) (CP2191) antibodies were purchased from ECM Biosciences. Rabbit polyclonal anti-CRMP2 (T514) (ab62478), mouse monoclonal anti-β-actin (A5441), and mouse monoclonal anti-α-tubulin (T9026) antibodies were purchased from Sigma. Mouse monoclonal anti-PTP4A3 (sc-130355) was purchased from Santa Cruz Biotechnology. Mouse monoclonal anti-GFP (11814460001) was purchased from Roche. Rabbit polyclonal anti-cofilin (3318) and Rabbit polyclonal anti-cofilin (S3) antibodies were purchased from Cell Signaling. The GST-tagged PTP4A3 and C104S were generated by PCR amplification and then ligated into the pGEX-4 T-2 expression vector.

### Phosphoprotein enrichment and two-dimensional (2D) gel electrophoresis

Phosphorylated proteins were purified from OCM-1 cells using the PhosphoProtein purification kit from Qiagen following the manufacturer’s instructions. Total protein extract (2.5 mg) was loaded onto PhosphoProtein affinity purification columns and eluted fractions with high concentrations of phosphorylated proteins were pooled, concentrated, and processed using a 2D clean-up kit (GE Healthcare), following the manufacturer’s protocol. Phosphorylated proteins were separated on their isoelectric point. Cleaned samples (200 µg) were incubated with IPG (immobilized pH gradient) strips, containing a linear 4–7 pH gradient (BIO-RAD), at room temperature for 16 h and isoelectric focusing performed at 50 μA, until 15,000 V/h was obtained, using a PROTEAN i12 IEF Cell at room temperature. For the second-dimension electrophoresis, the IPG strips were incubated for 10 min at room temperature in equilibration buffer (0.375 M Tris-HCl pH8.8, 6 M urea, 2% SDS, 4% glycerol) containing 130 mM DTT, and then for 10 min in equilibration buffer containing 135 mM iodoacetamide. The IPG strips were then placed at the top of a second gel, followed by SDS-PAGE, and Western blotting performed.

### GFP-trap

Cells were lysed in lysis buffer containing 10 mM Tris-HCl pH 7.5, 150 mM NaCl, 0.5 mM EDTA, 0.5% NP40, PhosSTOP (Sigma), and protease inhibitor cocktail (Sigma) and processed as previously published^15^.

### Immunoprecipitation

Cell lysate: Cells were lysed in 50 mM Tris-HCL pH 8, 150 mM NaCl, 5 mM EDTA, 2% NP40, PhosSTOP (Sigma), and protease inhibitor cocktail (Sigma).

Five milligrams of total protein extract was added to washed protein A sepharose and incubated 2 h at 4 °C for pre-clearing. After centrifugation, the supernatant was collected to eliminate non-specific binding and incubated overnight at 4 °C with anti-CRMP2 antibody at 1/200. Samples were incubated 4 h at 4 °C with 200 µl protein A Sepharose, previously washed using the same buffer as for the pre-clearing. After washing proteins were eluted with 0.1 M glycine pH 3 in three successive fractions. The presence of CRMP2 and its phosphorylation state (T514) were verified by Western blot.

### GST fusion protein purification

*Escherichia coli* (BL21) were used to produce the GST fusion proteins. To induce production, 1 mM isopropyl-β-D-1-thiogalactopyranoside (IPTG) was added to the culture 3 h prior to lysis. Bacteria were lysed by suspending in 50 mM Tris HCl, 250 mM NaCl, 2 mM DTT, 0.5 mM PMSF, and protease inhibitor cocktail (Sigma) and then were sonicated. The GST fusion proteins were purified from bacterial lysates with glutathione Sepharose 4B beads (Glutathione Sepharose 4B GST-tagged protein purification resin, GE Healthcare) in the lysis buffer overnight at 4 °C.

### Phosphatase activity test

A fraction of the GST fusion proteins was used to perform an *in vitro* phosphatase activity test. GST fusion proteins were washed in 50 mM Tris HCl pH 8, 140 mM NaCl, 2.7 mM KCl and 2 mM DTT. 3-O-Methylfluorescein phosphate (OMPF) substrate was added to the GST fusion proteins to a final concentration of 1 mM and samples were incubated 30 min at 37 °C in the dark. The absorbance at 450 nm was measured.

### GST pull-down

Fractions containing phosphorylated CRMP2 were pooled and added to the GST fusion proteins in 50 mM tris-HCl pH 8, 150 mM NaCl, 1 mM EDTA, 0.4% NP40, 2 mM DTT, and protease inhibitor cocktail (Sigma). Samples were incubated at 4 °C overnight and washed five times. The presence of CRMP2 and its phosphorylation state (T514) were verified by Western blot.

### Two-Dimensional (2D) random cell migration assayed by time-lapse video microscopy

Cells were seeded on a 50 µm/mL collagen I (BD Corning) matrix and migration was monitored by time-lapse video microscopy every 4 min for 12 h at 37 °C, as described in^7^.

### Flow Cytometry

Cells were seeded on a collagen I matrix, incubated for 24 h, serum starved for an additional 24 h, and dissociated using a non-enzymatic dissociation solution (Sigma-Aldrich). The amount of MMP14 on the cell surface was assessed as previously published^15^.

### Drug treatment

The inhibition of ROCKII in OCM-1 cells was achieved by Y27632 (Sigma-Aldrich) treatment at 10 μM for 24 h at 37 °C.

### Immunofluorescence

OCM-1 cells were seeded 24 h on glass-coverslips coated with 50 µg/mL collagen I. Cells were rinsed with PHEM buffer and fixed with 4% PFA/0.02% glutaraldehyde in PHEM (Pipes 120 mM, Hepes 50 mM, EGTA 20 mM, AcMg 4 mM in H_2_O) at 37 °C for 12 min. Cells were permeabilized in 0.5% Triton X-100 in PBS, blocked in 1% BSA, and then labeled with Acti-stain 555 phalloïdin (PHDH1, Cytoskeleton) at a dilution of 1/200 for 30 min at 37 °C. Cell nuclei were stained with DAPI and coverslips were mounted in 50% Glycerol/100 mg/mL DABCO (Sigma-Aldrich) in PBS. Images were acquired with Metamorph software using a 63X or 40X oil immersion objective of a wide-field microscope (DM RXA, Leica), equipped with a charge-coupled device camera (CoolSNAP HQ, Photometrics) and a piezoelectric translator at the base of the objective. Stacks of images were taken in the z axis with an interval of 0.2 nm for deconvolution. Deconvoluted images were processed using ImageJ.

Quantification: we performed a maximum intensity z projection of whole stacks. Objects with a size between 0 and 40 adjoining pixels were counted using segmentation by thresholding and granulometry functions of ImageJ and counts were then normalized to cell area.

### Chorioallantoid membrane (CAM) assay

We used 0.25 × 10^6^ cells to inoculate the CAM of fertilized chick eggs (EARL Morizeau, Dangers, France) as previously published^15^.

### Microrheology

Active microrheology, based on optical tweezers and fast confocal microscopy, was described previously^45^. Briefly, 2-µm diameter fluorescent microspheres (Thermo Fisher Scientific) were internalized in cells overnight. The culture medium was supplemented with 20 mM Hepes before the experiment, which was performed on a Nikon A1R confocal microscope equipped with a 37° incubator, a nanometric piezostage (Mad City Labs), and a home-made single fixed optical trap (1060–1100 nm, 2 W maximal output power, IPG Photonics). A bead was trapped by the optical tweezers (laser output power 1 W, corresponding to 150 mW for the sample). Following a 0.5-µm step displacement of the stage applied by the piezostage, the bead relaxation x_b_(t) towards the optical trap center was measured by single particle tracking. First, a model-independent phenomenological parameter, the rigidity index (*RI*), was calculated as the normalized area under the relaxation curve. *RI* values fall between 0 (very soft cytoplasm) and 1 (very stiff cytoplasm). The bead step displacement was defined as the initial displacement of the bead following the 0.5 µm step displacement of the stage. The values of the bead step displacement fall between 0 µm (very soft cytoplasm) and 0.5 µm (very stiff cytoplasm). Second, the Standard Linear Liquid (SLL) model^46,47^ was used to fit the relaxation curves. The SLL model consists of a Kelvin-Voigt body (a spring of elasticity μ and a dashpot of viscosity η in parallel) and a daspot in series. A custom-written Matlab code was used to fit the relaxation curves with the SLL model yielding the elasticity (in pN/µm) and viscosity (in Pa.s) of the bead microenvironment.

### Statistical analysis

Data are presented as the means ± SD for each condition except when indicated. Statistical analyses were performed using StatView software (SAS Institute Inc., Cary, NC, USA). The Mann Whitney-Wilcoxon test or Student’s t-test, when indicated, were used for comparisons.

## Supplementary information

supplemental figures
