# Supplementary material for: Protein tyrosine phosphatase 4A3 (PTP4A3/PRL-3) promotes the aggressiveness of human uveal melanoma through dephosphorylation of CRMP2
Source: Sci Rep. 2019 Feb 28;9:2990. doi: 10.1038/s41598-019-39643-y (PMC6395723; doi:10.1038/s41598-019-39643-y)
Supplement: Supplementary file 1 — supplemental figures [file 41598_2019_39643_MOESM1_ESM.pdf]

Title:

Protein tyrosine phosphatase 4A3 (PTP4A3/PRL-3) promotes the aggressiveness of human uveal melanoma through dephosphorylation of CRMP2.

Authors and affiliations:

Laura DUCIEL*[1,2], Océane ANEZO *[1,2], Kalpana MANDAL[3], Cécile LAURENT[4], Nathalie PLANQUE[1,5], Frédéric M. COQUELLE[1,2], David GENTIEN[6], Jean-Baptiste MANNEVILLE[7], and Simon SAULE[1,2].

Supplemental figures legend

a) Supplemental Figure 1: PTP4A3 expression and / or CRMP2 konckdown increases the percentage of cells with micronuclei in OCM-1 and MP41 cell lines

A) Visualization of nuclei and micronuclei in OCM-1 cells were assessed by DAPI staining after PFA fixation. Scale bar = 20 μm. Yellow arrows indicate micronuclei.

B) Quantification of the percentage of OCM-1 cells with micronuclei. Data are shown as the mean values +/- SEM. >150 cells were analyzed. **p < 0.01, ***p < 0.001, Student's t-test.

C) Visualization of nuclei and micronuclei in MP-41 cells were assessed by DAPI staining after PFA fixation. Scale bar = 20 μm.

D) Quantification of the percentage of MP-41 cells with micronuclei. Data are shown as the mean values +/- SEM. >150 cells were analyzed. **p < 0.01, Student's t-test.

b) Supplemental Figure 2: ROCKII inhibitor Y27632 decreases phosphorylated cofilin

Western blot showing the decrease of phosphorylated cofilin (S9) levels in OCM-1 cells treated with Y27632, confirming the inhibition of ROCKII. Twenty micrograms of protein extract was loaded. The detection of total cofilin was performed as a loading control.

c) Supplemental Figure 3: CRMP2 affects microtubules dynamics

A) Visualization of the microtubule network in OCM-1 cells as assessed by tubulin immunofluorescence after PFA fixation. Cells were treated with nocodazole and microtubule repolymerization followed for T0, 5, and 10 minutes after drug rinse. After deconvolution, images were processed using ImageJ64. Scale bar = 10 μm.

B) Quantification of the number of fragments of < adjoining 40 pixels normalized to the cell area in OCM-1 cells at T5. Data are shown as the mean values +/- SEM. ~20 cells were analyzed. *$p < 0.05$, Student's t-test.

C) Quantification of the percentage of cells with an aster at T5. Data are shown as the percentage +/- SD. ~20 cells were analyzed. **$p < 0.01$, *$p < 0.05$, Student's t-test.

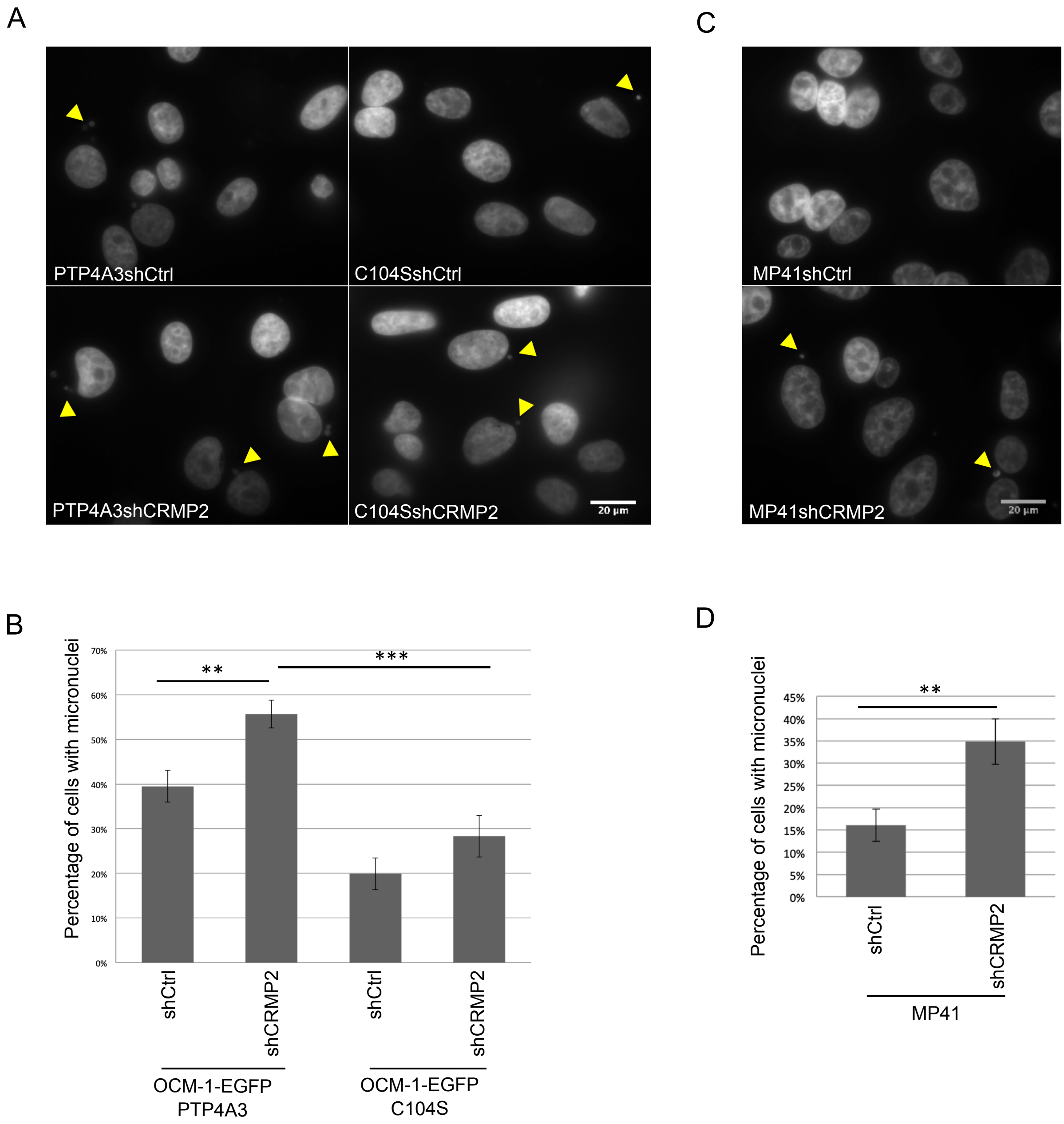

A
PTP4A3shCtrl
C104SshCtrl
PTP4A3shCRMP2
C104SshCRMP2
20 µm
C
MP41shCtrl
MP41shCRMP2
20 µm
B
Percentage of cells with micronuclei
70%
60%
50%
40%
30%
20%
10%
0%
**
***
shCtrl
shCRMP2
shCtrl
shCRMP2
OCM-1-EGFP PTP4A3
OCM-1-EGFP C104S
D
Percentage of cells with micronuclei
45%
40%
35%
30%
25%
20%
15%
10%
5%
0%
**
shCtrl
shCRMP2
MP41

A

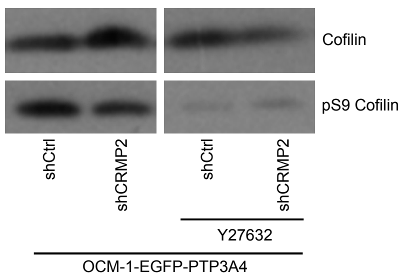
Cofilin
pS9 Cofilin
shCtrl
shCRMP2
shCtrl
shCRMP2
Y27632
OCM-1-EGFP-PTP3A4

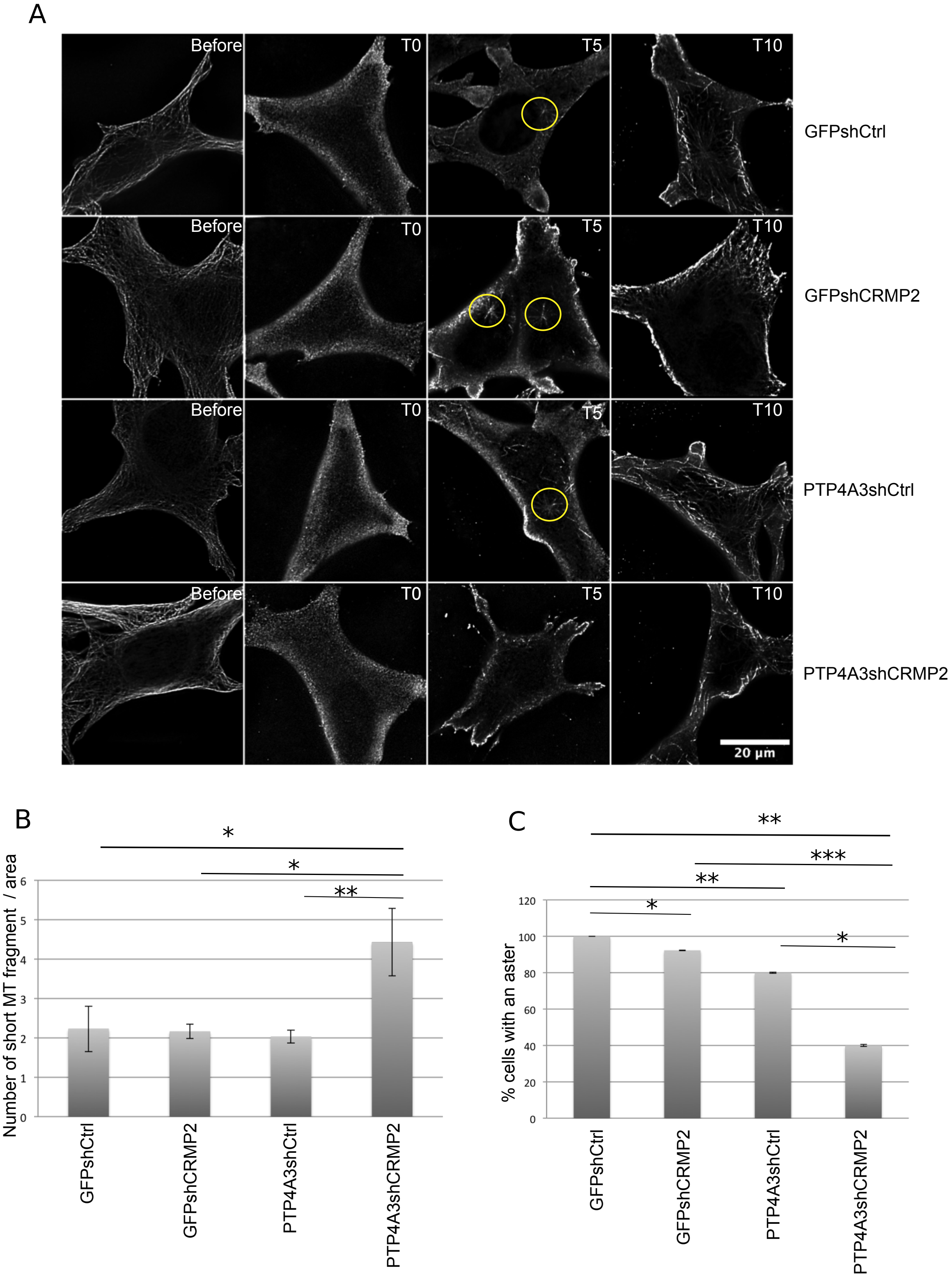
A
Before
T0
T5
T10
GFPshCtrl
GFPshCRMP2
PTP4A3shCtrl
PTP4A3shCRMP2
20 µm
B
Number of short MT fragment / area
0
1
2
3
4
5
6
*
*
**
GFPshCtrl
GFPshCRMP2
PTP4A3shCtrl
PTP4A3shCRMP2
C
% cells with an aster
0
20
40
60
80
100
120
**
***
**
*
*
GFPshCtrl
GFPshCRMP2
PTP4A3shCtrl
PTP4A3shCRMP2
